# Accurate In-Vivo Quantification of CD19 CAR-T Cells after Treatment with Axicabtagene Ciloleucel (Axi-Cel) and Tisagenlecleucel (Tisa-Cel) Using Digital PCR

**DOI:** 10.3390/cancers12071970

**Published:** 2020-07-20

**Authors:** Anita Badbaran, Carolina Berger, Kristoffer Riecken, Anne Kruchen, Maria Geffken, Ingo Müller, Nicolaus Kröger, Francis A. Ayuk, Boris Fehse

**Affiliations:** 1 Department of Stem Cell Transplantation, University Medical Centre (UMC) Hamburg-Eppendorf, 20246 Hamburg, Germany; badbaran@uke.de (A.B.); su.berger@uke.de (C.B.); k.riecken@uke.de (K.R.); n.kroeger@uke.de (N.K.); 2 Paediatric Stem Cell Transplantation and Immunology, UMC Hamburg-Eppendorf, 20246 Hamburg, Germany; a.kruchen@uke.de (A.K.); i.mueller@uke.de (I.M.); 3 Institute for Transfusion Medicine, UMC Hamburg-Eppendorf, 20246 Hamburg, Germany; Maria.Geffken@uke.de

**Keywords:** tisagenlecleucel (tisa-cel, Kymriah), axicabtagene ciloleucel (axi-cel, Yescarta), digital PCR, CAR-T cell monitoring, CAR-T cell persistence

## Abstract

Immunotherapy with CD19-specific chimeric antigen receptor (CAR-) T cells has shown excellent efficacy in relapsed/refractory B-cell cancers. The in vivo expansion and persistence of CAR-T cells after infusion are important response- and toxicity-determining variables, but diagnostic tools are largely missing. We showed previously for axi-cel that digital PCR (dPCR) is excellently suited to monitoring CAR-T cells in vivo. Here, we aimed to develop an analogous dPCR assay for tisa-cel. To do so, we cloned and sequenced the CAR construct from the lentiviral tisa-cel vector and designed primers and Black hole quencher (BHQ) probes complimentary to sequences present in the FMC63 scFv part of axi-cel (assay A), tisa-cel (T), and both constructs (U = “universal”). In conjunction with excellent specificity, all assays have a detection limit of one single CAR copy, corresponding to a sensitivity of approximately 1 in 5000 cells (0.02%) for 100 ng genomic DNA (for one vector copy per transduced cell). The new universal assay was first validated using patient samples previously quantified with the axi-cel-specific dPCR and thereafter applied to quantify and monitor adoptively transferred axi-cel and tisa-cel T cells in post-infusion samples (peripheral blood, bone marrow, liquor, and ascites). Actual CAR-T counts per µl were calculated, taking into account vector copy and peripheral blood mononuclear cell (PBMC) numbers, and showed very good correlation with flow cytometry results. We conclude that our novel dPCR assay is optimally suited to monitoring tisa-cel and axi-cel CAR-T cells in real-time in various body fluids.

## 1. Introduction

The general principle of chimeric-antigen-receptor (CAR-) T cells was first described in the late 1980s/early 1990s [1,2]. Their clinical breakthrough required several improvements, particularly the introduction of costimulatory signals, resulting in so-called second-generation CARs [3,4]. Eventually, CAR-T cells targeting CD19 demonstrated excellent efficacy in otherwise refractory late-stage B-cell malignancies [5,6]. Since then, hundreds of clinical studies with many different CARs against a variety of antigens have been performed to treat hematological malignancies but also solid tumors [4]. Importantly, two CD19-CAR-T cell products (tisagenlecleucel/tisa-cel/Kymriah and axicabtagene ciloleucel/axi-cel/Yescarta) have been licensed in many countries [4], and a number of other CAR-T products are in late-phase clinical testing.

Several reports have demonstrated the importance of CAR-T cell engraftment, expansion, and persistence in vivo for clinical outcomes after axi-cel treatment [7,8]. In the first 28 days, CAR-T cell peak concentrations and area-under-the-curve (AUC) values have been associated with long-term efficacy [7,8]. In contrast, no association between AUC and efficacy was reported for tisa-cel in acute and chronic B-cell leukemia (B-ALL and B-CLL, respectively), but vector-copy numbers per µg DNA rather than actual CAR-T cell numbers per µl blood were determined [9,10]. Moreover, both tisa-cel studies found a direct link between clinical response and persistence of tisa-cel in peripheral blood [9,10]. Together, the available data underline the importance of reliably and accurately quantifying CAR-T cells in vivo to obtain dependable real-world data for the two approved products. Unfortunately, precise and fast assays are largely missing.

Similarly to CARs, the principles of digital PCR (dPCR) were described in the early 1990s, but the technique became broadly used only in the last decade, facilitated by the introduction of easy-to-use dPCR devices [11,12,13]. In short, by partitioning a polymerase chain reaction into large numbers (e.g., 20,000) of individual PCRs, digital PCR uses the limiting dilution principle, where each individual partition either contains the PCR target sequence (“1”) or does not contain it (“0”). As in quantitative PCR, amplification of the target sequence is detected based on fluorescence, but this is carried out individually for each partition; absolute numbers of target copies in a given sample are calculated by applying Poisson correction [11,12,13]. Depending on the number of fluorescence channels, two or even more dPCRs can be performed for single samples (“multiplexing”). The excellent sensitivity, specificity, and reproducibility of dPCR make this method particularly useful for the precise quantification of rare events, not the least in clinical diagnostics [13]. We have recently developed a dPCR assay that facilitates the rapid, sensitive, and accurate quantification of axi-cel CAR-T cells in various body fluids [14]. We here describe the development, testing, and clinical application of novel dPCRs, including one assay detecting the original sequence of the single-chain fragment derived from the murine anti-human CD19 antibody FMC63 [15]. FMC63 targets a specific epitope of CD19 found in exon 4 of the CD19 gene [16]. We show that this novel dPCR shows excellent performance in quantifying both axi-cel and tisa-cel CAR-T cells.

## 2. Results

### 2.1. Development and Initial Testing of Digital-PCR Assays

Whereas tisa-cel and axi-cel are based on different vector backbones and CAR designs, they have been known to contain the same CD19-targeting single-chain fragment FMC63 [5,7]. However, it remains to be verified whether protein identity is also true on the DNA level. In fact, codon-optimization is often applied to improve transgene expression in gene therapy. Moreover, due to the nature of retroviral vectors, single point mutations are frequently observed in their sequences. Obviously, any sequence differences would be highly relevant for amplicon design.

To address this point, we first obtained the full-length sequence of the tisa-cel CAR by PCR amplification and Sanger sequencing. We indeed found parts of complete identity to the sequence of the axi-cel vector previously published by Kochenderfer et al. [17], but we also found some relevant differences between the two vectors: (1) different signal peptides have been utilized, (2) the linkers between the two halves of the FMC63 scFv are different, (3) as expected, tisa-cel contains the 4-1BB domain (instead of CD28 in axi-cel), (4) a point mutation within CD3 ζ was identified in tisa-cel, leading to the exchange of one amino acid (257C→A relative to CD3 ζ mRNA NM_000734.4, resulting in a Gln→ Lys substitution). 

**Figure 1:**
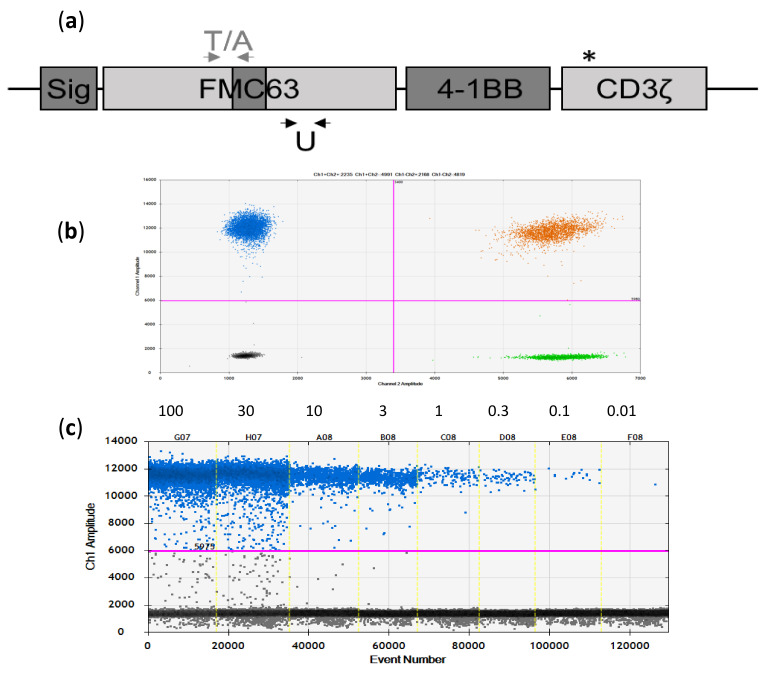
Novel FMC63-directed digital PCR (dPCR) assays for tisa-cel and axi-cel. (**a**) Scheme of the CAR cDNA used in the two vector constructs (tisa-cel and axi-cel). Identical sequences are indicated in light grey, different sequences in dark grey (Sig = signal peptide). In the axi-cel CAR, the CD28 co-stimulation domain is used instead of the 4-1BB domain shown in the picture. The CD3ζ domain differs by one point mutation, indicated by the asterisk (see main text for details). Localization of the tisa-cel (T) and axi-cel (A) specific amplicons/primers is depicted by grey arrows above the graphic, whereas localization of the universal (U) primers is indicated by the black arrows below the plot. Not to scale. (**b**) Excellent separation of negative and positive signals in the two-dimensional plot as exemplified for the universal assay (*Y*-axis: CAR-specific signals, *X*-axis: HCK reference (REF) signals). (**c**) Dilution series of axi-cel product tested with the U assay.

Using this information, we decided to design and apply a universal (U) amplicon for both axi-cel and tisa-cel located in the FMC63 part of both vectors. As controls, we also devised two further amplicons, each in the FMC63 part specific for tisa-cel (T) and axi-cel (A), respectively. Amplicon U has a length of 107 bp, whereas both amplicons T and A are 95 bp long (Figure 1a). 

To assess the principal usefulness of the three assays, we tested them on CAR-positive and CAR-negative samples. All three assays demonstrated very good separation of positive and negative droplets, as exemplarily shown for amplicon U in Figure 1b,c.

### 2.2. Assay Specificity, Sensitivity, and Reproducibility

We went on to evaluate the specificity of the assays. To this end, we ran the assays on two mixes of genomic DNA from healthy donors (each mix consisted of gDNA from 10 different buffy coats). All tests were negative. Moreover, all pre-infusion samples and the zero values of serial dilutions (see below) as well as non-template controls were negative in all performed analyses. In summary, we did not detect a definitive limit-of-blank, thus confirming excellent specificity of the dPCR assays introduced here.

**Figure 2:**
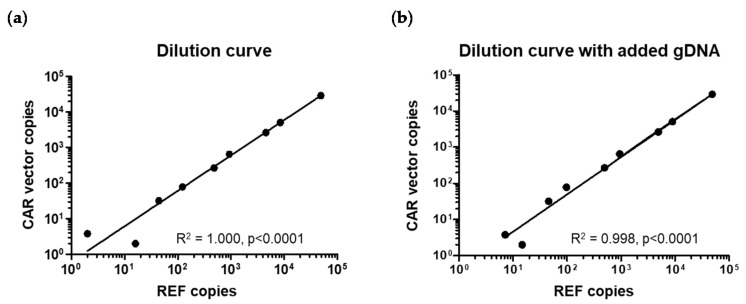
Highly significant correlation of CAR and REF dPCRs and excellent sensitivity. (**a**) Assessment of a dilution series gDNA sample containing a mean of three axi-cel vector copies per cell. (**b**) The same dilution series as in (a) was analyzed, this time in the presence of 100 ng third-party gDNA as a potentially disturbing factor.

We then tested the sensitivity of the novel assays, first on an axi-cel product following our previous work [14]. To do so, we diluted a sample containing a mean of approximately three axi-cel copies per cell (Figure 1c). Dilutions were either performed in water or in third-party genomic DNA to address the potential impact of DNA amount on sensitivity. At the highest concentration, we assessed approximately 100 ng of gDNA, corresponding to 30,000 HCK and 45,000 axi-cel copies. Thus, at the lowest dilution (10^−4^) tested, the sample contained the equivalent of three haplogenomes, the theoretical threshold for reproducible detection. We applied the U and A assays capable of detecting the axi-cel sequences. For control, the dilution series was also measured using our previously established axi-cel dPCR [14]. As shown in Figure 2a,b for the universal assay, we found a highly significant correlation (R^2^ = 1.000, *p* < 0.0001 without; R^2^ = 0.998, *p* < 0.0001 with added third-party DNA) between the numbers of HCK and axi-cel copies up to the lowest concentration. The data were very similar for the novel A and, as expected, the established axi-cel assays.

In order to evaluate the sensitivity of the tisa-cel specific assays, we made use of a patient sample containing approximately one CAR copy per 100 cells or 200 HCK reference (REF) copies. Starting with 150 ng gDNA (corresponding to ca. 45,000 REF and thus 225 tisa-cel copies), we used three-fold dilutions. We performed dPCRs with both the universal and the tisa-cel specific assays, each in duplex. Again, we observed a very good linear correlation between REF and tisa-cel CAR signals down to 81-fold dilutions that were positive in all four assays (2 × tisa-cel specific, 2 × universal). Since an 81-fold dilution would be expected to contain approximately three tisa-cel copies, this observation was in perfect accord with the theoretical detection threshold.

We next studied the reproducibility of assay U. To do so, we repeated the initial measurements performed on individual CAR-positive samples with the established axi-cel assay three times in independent setups (I–III), each time in duplicate (a,b). The obtained data summarized in Table 1 confirm excellent reproducibility with very low intra- and inter-assay variation. 

### 2.3. Analysis of Patient Samples

**Figure 3:**
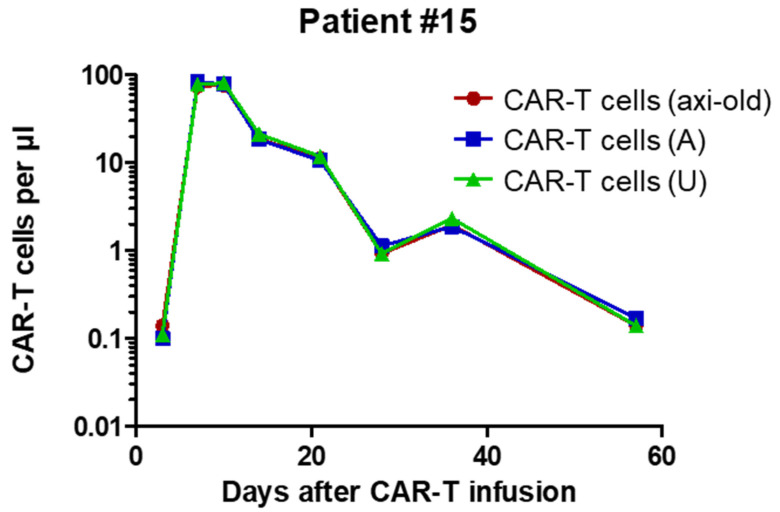
Kinetics of CAR-T cells in axi-cel patient #015 as assessed by the previously reported axi-cel (axi old) assay in comparison with the new FMC63-directed axi-cel (A) and universal (U) assays. As evidenced here, results from all three assays are essentially identical. Correlation coefficients and corresponding *p* values based on Pearson statistics are indicated.

The data presented in Table 1 not only confirm the outstanding reproducibility but also indicate the perfect agreement between numbers obtained with the new universal assay and the previously established axi-cel specific dPCR [14]. To further cross-validate the two dPCR assays, we used material from another patient (#015) treated with axi-cel, for whom several follow-up samples were available. We performed dPCRs on eight consecutive peripheral blood mononuclear cell (PBMC)-derived gDNA samples from this patient. As previously described [14], the mean vector copy number (mVCN) had been determined on the axi-cel product and was used to calculate numbers of axi-cel CAR-T cells in the individual samples of the patient based on dPCR data independently obtained with the two different assays. Importantly, data obtained with both the axi-cel specific and the universal dPCR assays were in perfect accord (Figure 3).

In the next step, we used the novel U assay to prospectively investigate samples of consecutive patients (#020, #021, #024, #027) treated with tisa-cel. Based on the information provided by the manufacturer, tisa-cell products usually contain only one vector copy per cell, which is in clear contrast to axi-cel. Nevertheless, we used the provided data on transduction rate and copy numbers to calculate the mVCN (Table 2), applying the Poisson formula and the scheme previously described [14]. The mVCN was in turn utilized to calculate actual numbers of CAR-T cells per µl blood.

**Figure 4:**
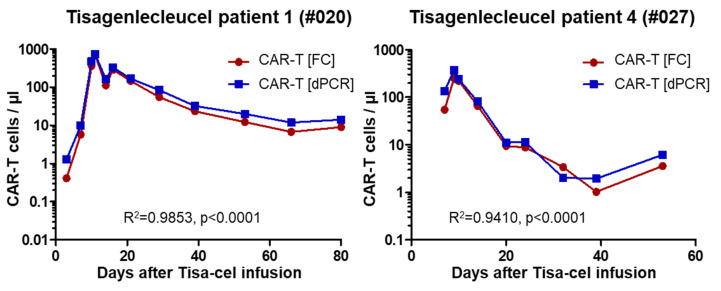
Comparison of dPCR and flow cytometry (FC) data for the two pediatric tisa-cel patients.

For the two pediatric patients, flow-cytometry analyses were performed independently and in a blinded way, and data were only merged for the figure. Figure 4 shows flow cytometry and dPCR data obtained with the universal assay for these two pediatric tisa-cel patients (#020, #027, see Table 2). As evident, CAR-T cell numbers as determined with the two different methods are in excellent accord with, in most cases, slightly higher values determined by dPCR. These data underline the usefulness of the universal assay for the follow-up of tisa-cel treated patients.

Finally, we aimed to test the applicability of the universal assay to quantify CAR-T numbers in body fluids other than blood. To do so, we measured gDNA samples previously isolated from liquor (*n* = 4), ascites (*n* = 1), bone marrow (*n* = 3), and tumor (lymph-node) biopsies (*n* = 2) from nine different axi-cel patients, and we determined CAR-T numbers per µl or per million cells (Table 3). The obtained values were essentially identical to those previously acquired for these samples using the established axi-cel assay (Table 3), again underlining the robustness of the dPCR assays. 

## 3. Discussion

After the initial clinical breakthrough with CD19-directed CAR-T cells in CLL in 2011 [5], this novel type of adoptive immune effector cellular therapy has become a topic of huge interest and fast development in hematology and oncology [4,18]. Large clinical studies, particularly in B-cell malignancies (e.g., [7,8,9,10]), resulted in the approval of the first CD19-CAR products, namely tisagenlecleucel/tisa-cel/Kymriah and axicabtagene ciloleucel/axi-cel/Yescarta. Moreover, several other CAR-T products targeting CD19 as well as other antigens are in late clinical testing. In parallel, the CAR technology is being steadily optimized, as evidenced by novel constructs with improved performance and/or safety features such as third- and fourth-generation CARs (the latter also referred to as TRUCKs) or bi-specific CARs [4,18].

In contrast to conventional medicines, cellular therapies represent living drugs, i.e., their clinical efficacy is directly dependent on their engraftment, expansion, and functional persistence. In fact, lack of engraftment as well as loss of CD19 CAR-T cells have directly been linked with disease progression and relapse, respectively [7,8,9,10]. Interestingly, discordant data were reported for axi-cel [7,8] and tisa-cel [9,10] regarding a possible influence of the degree of CAR-T cell expansion over time (area under the curve) on the likelihood of tumor response. This discrepancy, which might, amongst others, be due to differences in CAR constructs and/or small sample numbers investigated underlines the necessity of obtaining additional real-world data to better understand the mode of action and eventually improve the therapeutic efficacy of CAR-T cells for those patients who so far do not benefit. 

In light of the above, there is an urgent need for reliable in-vivo data on CAR-T cell numbers to study pharmacodynamics and pharmacokinetics in individual patients. At the same time, despite the commercialization of tisa-cel and axi-cel, suitable assays to enumerate CAR-T cells have largely been missing. More recently, a flow cytometry based “CD19 CAR detection reagent” has become commercially available from Miltenyi Biotec (Bergisch Gladbach, Germany). In addition, qPCR-based methods have been developed [19].

We reasoned that digital PCR is ideally suited to detecting and quantifying CAR-T cells in vivo. Earlier this year, we introduced an axi-cel specific assay [14]. In the current work, we determined the full CAR-coding sequence of the lentiviral vector used in tisa-cel. We then performed a sequence comparison of the tisa-cel and axi-cel CD19-directed CAR cDNAs and identified unique as well as common sequence stretches. Based thereon, we designed three novel amplicons for dPCRs located in the FMC63 region of the two CARs (U = universal, T = tisa-cel, and A = axi-cel specific). We show that all three assays enable the quantification of CAR-T cells in artificial cell mixtures but, more importantly, in patient peripheral blood samples. The “universal” assay allows for the quantification of both tisa-cel and axi-cel CAR-T cells and therefore might be most useful. In consequence, subsequent broad testing was performed for this assay only.

We found that the universal assay combined excellent specificity (no positive signal observed in multiple samples from non-treated individuals) with the highest possible sensitivity (detection of single copies). These features make the assay ideally suited to diagnostic purposes. In fact, in our experience, the assay ensures the detection of CAR-T cells at concentrations as low as 1–2 axi-cel or 2–3 tisa-cel per 10,000 blood cells in routine use (i.e., with ca. 100 ng gDNA, corresponding to 15,000 diploid cells). The difference is due to the observed higher mVCNs in axi-cel as compared to tisa-cel products. If higher sensitivities are required, the amounts of gDNA for testing could be increased and/or sorted cell populations could be used. We also have provided evidence of the applicability of our assay to gDNA obtained from ascites, cerebrospinal fluid, and tumor biopsies. 

For two of our tisa-cel patients, we have been able to compare CAR-T cell numbers as assessed by the universal dPCR to data independently determined by flow cytometry. Importantly, the results obtained with the two different methods were in excellent accord, although, in most cases, slightly higher numbers were found by dPCR (Figure 4). The latter finding could most probably be explained by integrated, but not expressed, transgene copies, a common observation with retroviral vectors. The variance was most pronounced at the earliest time point, i.e., up to day 7 post infusion. Since DNA was obtained from whole blood in these patients, it is tempting to speculate that the higher signals in dPCR are due to cell-free DNA floating as a result of early cell death after infusing the thawed cell product, but this needs empirical confirmation. Overall, the observed almost perfect correlation between the two independent methods, flow cytometry and digital PCR, is very important, since the two methods in many regards complement each other. Flow cytometry determines actual numbers of CAR-expressing cells and permits their phenotypic characterization, but it depends on fresh and intact cells. DPCR, on the other hand, allows us to determine vector copy numbers and will still function with old or even dead cells. Sample quality might become particularly relevant in outpatient settings when prolonged transportation is required due to a patient living in another city. Indeed, overnight transport might have influenced results for the last three probes of the second pediatric tisa-cel patients (compare Figure 2). Besides its robustness, dPCR potentially facilitates higher sensitivity; depending on the actual numbers of tested cells, the latter might be 1–2 logs higher than that of flow cytometry, at much lower costs. However, the combined use of both methods to detect CAR-T cells might be particularly desirable, when subsequent treatments, e.g., with checkpoint inhibitors, depend on definitive evidence for the presence of remaining CAR-T cells. 

Altogether, we have introduced and tested a novel “universal” dPCR assay that facilitates the enumeration of CAR-T cells in patients treated with the commercial CAR-T products (tisa-cel or axi-cel). We envisage that the sensitive in-vivo monitoring of CAR-T cells in treated patients will help to improve adoptive immunotherapy in the future.

## 4. Materials and Methods

### 4.1. Identification of Primers and Probes for Digital PCR

For PCR amplification of the complete cDNA of the tisa-cel vector region containing the CAR, primers were designed by educated guess to bind in front of the CAR’s cDNA (EF1alpha-Forward 5′-GGTGGAGACTGAAGTTAGGCC) and behind the CAR’s cDNA in reverse orientation (wPRE-Reverse 5′-GCAATGCCCCAACCAGTG). The obtained PCR fragment was sequenced using the standard Sanger method (Eurofins Genomics, Ebersberg, Germany) with the indicated forward primer, and subsequently an additional internal primer (5′-CAGCCATTTACTACTGTGCCAA) was designed based on the first obtained sequence. 

Primers and probes for three different amplicons (U, T, A), all located in the FMC regions of the CAR construct, were designed using PrimerExpress_3.0.1 (ThermoFischer, Kandel, Germany). One amplicon was universal (U) for both tisa-cel and axi-cel, whereas the other two were designed to be tisa-cel (T) and axi-cel (A) specific by locating the reverse prime in the different linkers present in the two vector constructs (Figure 1a). The universal primer/probe combination (U) is available from Bio-Rad (Foster City, CA, USA) as an Expert Design Assay (cat. number dEXD88164642).

### 4.2. Genomic DNA and dPCR

Genomic DNA (gDNA) preparation from whole peripheral blood (PB) and PBMC and droplet-digital PCR were performed as recently described [14]. 

As previously [14], we performed duplex PCRs to simultaneously amplify CAR- and reference-gene (REF) sequences. BHQ probes labeled with FAM (CAR) and HEX (REF) were used. In one set of experiments, we applied the RPP30 reference assay from Bio-Rad. Standard concentrations of primers (900 nM) and probes (250 nM), as suggested for dPCR by Bio-Rad, were used. We typically tried to analyze at least 100 ng gDNA, corresponding to approximately 15,000 diploid genomes (cells) and thus 30,000 copies of any diploid genes per sample. To reduce sample viscosity and improve target accessibility we incubated the reaction mix in the presence of 5 U EcoRI (Thermo Fischer) or 25 U HaeIII (New England Biolabs, Frankfurt a.M., Germany) at room temperature for 5 min before cycling. We routinely use sequences located in the diploid hematopoietic cell kinase (HCK) gene and the (male-specific) haploid DFFRY gene as REF [14,20], but any other REF not interfering with the CAR assay and not harboring the target sequence of EcoRI could also be used. Droplets were analyzed with the QX100 droplet reader, and data were processed with QuantaSoft_v1.7 software (Bio-Rad) that performs automatic Poisson correction [14].

### 4.3. Patients and Patient Material

Samples from two adult axi-cel (CAR#015, #016) and four consecutive tisa-cel patients (CAR#020, CAR#023, CAR#024, CAR#027; 2 children, 2 adults) who received treatment between March 2019 and April 2020 were included in analyses with written informed consent. Characteristics of included patients are summarized in Table 1. We also used material from nine patients treated with axi-cel (#005, #006, #010, #011, #013, #014, #015, #016, #023); their characteristics will be published elsewhere. Our study was approved by the local ethics committee (#PV7081, #PV5777). 

Peripheral blood mononuclear cells (PBMC) were isolated from patient blood (bone marrow, liquor) by Ficoll gradient centrifugation using SepMate (Stem Cell Technologies, Cologne, Germany), as described [14]. Alternatively, DNA was prepared from whole blood.

To calculate PBMC numbers in a given sample, differential blood counts were determined using standard automated blood cell counters (Advia 2120i (Siemens, Erlangen, Germany), ABX Pentra XL80 (Horiba, Irvine, CA, USA)). PBMC were determined as WBC minus granulocytes (ANC) and/or lymphocytes + monocytes. At low WBC, blood cell populations were counted manually.

### 4.4. Flow Cytometry

To determine total CD3+ T cells, 100 µl peripheral blood (EDTA) was stained with anti CD45 VioGreen, anti CD3 VioBlue, and 7AAD, according to the manufacturer’s instructions (Miltenyi Biotec). Whole blood was lysed with BD Pharm Lyse™ solution (BD Biosciences, Heidelberg, Germany) and subsequently analyzed on a MACSQuant10 Analyzer (Miltenyi Biotec). Total CD3+ T cell numbers were calculated from WBC counts, determined using standard automated blood cell counters. 

CD19 CAR expressing T cells were determined using CD19 CAR detection reagent (Miltenyi Biotec), according to the manufacturer’s instructions. In brief, 1 mL peripheral blood (heparinized) was bulk lysed using BD Pharm Lyse™ solution, washed with FACS buffer (PBS containing 2% FBS), and resuspended in 100 µl FACS buffer. Cells were stained with CD19 CAR Detection Reagent for 10 min at room temperature, washed with FACS buffer, and stained with anti CD45 VioGreen, anti CD3 VioBlue, anti CD4 PerCPVio700, anti CD8 APC, anti CD19 FITC, and anti-biotin PeVio770 (Miltenyi Biotec) for 10 min at room temperature. After washing with FACS buffer, cells were resuspended in 500 µl FACS buffer and subsequently analyzed on a MACSQuant10 Analyzer. Routinely, at least 125,000 cells were analyzed in the lymphogate to ensure high sensitivity. 

### 4.5. Calculations and Statistics

To assess mean vector copy numbers (mVCNs) in a given axi-cel or tisa-cel product, duplex dPCRs were performed with specific and REF primers, as described [14]. Copy numbers were calculated for the two used amplicons with QuantaSoft_v1.7 software. REF copy numbers indicated absolute numbers of alleles (and, divided by 2, cells) in the sample. Based thereon, data from CAR-specific dPCR were used to calculate absolute numbers of vector copies in the given product. Importantly, CAR-T product represents a mixture of transduced and non-transduced cells, which needs to be considered to correctly calculate the actual mVCN per transduced cells. As described previously [14], for the sake of convenience, we assumed that all cells are equally susceptible to transduction by the used retroviral vectors, and, based thereon, we applied Poisson statistics to determine the transduction rate based on the measured VCN [21]. Finally, by dividing the measured VCN in the product by the calculated transduction rate, we obtained the mVCN per transduced cell [14]. 

We applied two-tailed Pearson statistics with a confidence interval of 95% to determine correlation coefficients. For statistical analyses, GraphPad Prism (San Diego, CA, USA) was used.

## 5. Conclusions

The novel “universal” digital PCR assay is excellently suited to the high sensitivity, precise, and reproducible detection of tisa-cel as well as axi-cel CAR-T cells in vivo. Using our proposed method, the actual numbers of CAR-T cells per µl body fluid can be determined. Close monitoring of CAR-T cell numbers in vivo is of the utmost importance to guide subsequent interventions in individual patients, but it can also be expected to improve our general understanding of CAR-T therapies in real-world settings.

## Figures and Tables

**Table 1:** Reproducibility of the universal (U) assay.

Test Sample^1^	Axi-Cel Assay	Universal Assay							Mean
		Ia	Ib	IIa	IIb	IIIa	IIIb	RPP30	
		CAR-T Cells/µl Blood							
185	0.19	0.12	0.19	0.17	0.12	0.16	0.19	0.16	**0.16**
187	0.38	0.39	0.4	0.38	0.38	0.4	0.4	0.34	**0.38**
196	17.82	17.9	18.1	17.7	17.15	17.83	17.77	18.11	**17.80**
201	10.72	10.05	9.4	9.03	10.11	8.82	8.6	9.49	**9.53**
204	0.66	0.76	0.8	0.7	0.6	0.51	0.76	0.72	**0.69**
291^2^	0.04	0	0.1	0	0.13	0.03	0.06	0.16	**0.07**

^1^ All samples were from axi-cel patient #016. The first column shows the original data from her diagnostic dPCR, performed with the axi-cel assay [14]: I to III are three independent experiments, all performed in duplicate (a,b) with the universal assay and our REF gene; “RPP30” is an independent replication using the universal assay in conjunction with the RPP30 reference assay from Bio-Rad. Mean values over the eight independent measurements are indicated in the last column in **bold**. ^2^ For the tested amount of gDNA, a CAR-T cell concentration of 0.05 per µl blood corresponds to less than three vector copies per measured gDNA sample. In accord with Poisson distribution, the vector was therefore not detected in any individual test.

**Table 2:** Characteristics and data for patients treated with tisa-cel.

Patient	#020	#021	#024	#027
**Age (at CAR infusion)**	10	59	51	7
**Sex**	male	female	male	female
**Diagnosis**	BPC-ALL 1^st^ relapse	DLBCL 1^st^ relapse (early, refractory)	DLBCL 1^st^ relapse (early)	BPC-ALL 2^nd^ relapse
**Calculated mVCN**	1.103	1.095	1.0	1.093
**CAR-T peak/µl (day)**	743 (11)	25 (6)^1^	21 (8)	375 (9)

^1^ Patient died on day 6. Abbreviations: BPC-ALL: B progenitor cell ALL; DLBCL: diffuse large B-cell lymphoma; mVCN: mean vector copy number.

**Table 3:** Applicability of dPCR to different biological specimens.

Sample ID	Type of Material	CAR-T Cells per µl	
		Axi-Cel Assay	Universal Assay
116	Liquor cerebrospinalis (patient #010)	27.66	27.06
117^1^	Liquor cerebrospinalis (patient #010)	n.d.	27.31
133	Liquor cerebrospinalis (patient #013)	2.24	2.24
193	Liquor cerebrospinalis (patient #016)	32.76	33.09
139	Ascites (patient #006)	1.32	1.34
		**CAR-T Cells per Million Cells**	
26	Bone marrow (patient #005)	26,432	27,467
97	Bone marrow (patient #005)	27,780	28,670
324	Bone marrow (patient #023)	7991	8506
234	Tumor (lymph-node) biopsy (patient#011)	62,948	62,204
367	Tumor (lymph-node) biopsy (patient#014)	19,647	19,656

^1^ Samples 116 and 117 represent repeated cerebrospinal-fluid samplings from patient #010 on the same day. Abbreviation: n.d., not determined.
